# S168. AUDITORY TRANSCALLOSAL FIBERS AND AUDITORY HALLUCINATIONS

**DOI:** 10.1093/schbul/sby018.955

**Published:** 2018-04-01

**Authors:** Dean Salisbury, Yiming Wang, Brian Coffman

**Affiliations:** 1 University of Pittsburgh School of Medicine; 2 University of Pittsburgh

## Abstract

**Background:**

Auditory verbal hallucinations (AVH) are one of the most common symptoms in schizophrenia. Connectivity between left and right auditory cortex may be related to AVH. The aim of this study was to examine transcallosal auditory cortex connectivity in first-episode schizophrenia patients (FESz) who experience AVH.

**Methods:**

Diffusion spectrum imaging (DSI) data were obtained from 29 FESz and 23 healthy controls (HCs). Of the 29 FESz participants, 15 were AVH-, with a score of 0 for auditory hallucinations, voices commenting, and voices conversing measured with the Scale for the Assessment of Positive Symptoms (SAPS), and 14 were AVH+, with a score of 2 or greater on at least one of these questions. The three groups (AVH+, AVH-, and healthy controls) were matched for age, parental socioeconomic status, years of education, IQ, gender, and handedness. A deterministic fiber tracking algorithm was used to identify the transcallosal auditory white matter tract, which was identified as the 1000 fibers passing through the posterior third of the corpus callosum and ending bilaterally in Brodmann’s area 22, Heschl’s gyrus, or planum temporale. Transcallosal auditory cortex connectivity was compared between groups for tract volume, generalized Fractional Anisotropy (gFA), and isotropy.

**Results:**

MANOVA revealed a significant difference in connectivity between groups (F(6, 94) = 2.34, p = .038) that was driven by group differences in tract volume (F(2, 49) = 3.46, p =.039) and gFA (F(2, 49) = 4.77, p = .013)). Within FESz, AVH severity significantly correlated with auditory cortex transcallosal gFA (r =-.44, p =.013). Pairwise t-tests indicated lower gFA and greater tract volume for AVH+ vs AVH- (p’s < .05). HCs had a trend towards greater gFA (p = .068) vs AVH+ and tract volume (p = .063) vs AVH-. All other comparisons were nonsignificant (p >.1).

**Discussion:**

These findings suggest that structural connectivity differences may underlie AVH in schizophrenia, even early in disease course. FESz participants with AVH have less efficient transcallosal auditory connectivity compared to those without AVH. The reduced gFA in FESz correlated with hallucination severity, suggesting that inefficient coordination of left and right hemisphere auditory processing, crucial for language, was impaired in the disorder. The transcallosal structural integrity and connectivity may indicate a subtype characterized by AVH. Current work is determining the extent to which this fiber deficit is common across Kraepelinian diagnostic categories of psychosis (e.g., bipolar disorder and depression with psychotic features).

